# Integrated transcriptome and hormonal analysis of naphthalene acetic acid-induced adventitious root formation of tea cuttings (*Camellia sinensis*)

**DOI:** 10.1186/s12870-022-03701-x

**Published:** 2022-07-04

**Authors:** Yongxin Wang, Dandan Pang, Li Ruan, Jinbo Liang, Qiang Zhang, Yinhong Qian, Yazhen Zhang, Peixian Bai, Liyun Wu, Hao Cheng, Qingmei Cui, Liyuan Wang, Kang Wei

**Affiliations:** 1 Key Laboratory of Tea Biology and Resources Utilization, Ministry of Agriculture, National Center for Tea Improvement, Tea Research Institute Chinese Academy of Agricultural Sciences (TRICAAS), Hangzhou, 310008 China; 2 Tea Research Institute, Yunnan Academy of Agricultural Sciences, Menghai, 666201 China; 3 Tea Research Institute of Enshi Academy of Agricultural Sciences, Enshi, 445000 China

**Keywords:** Adventitious root, *Camellia sinensis*, Hormone, NAA, Transcriptome, WGCNA

## Abstract

**Background:**

Tea plant breeding or cultivation mainly involves propagation via cuttings, which not only ensures the inheritance of the excellent characteristics of the mother plant but also facilitates mechanized management. The formation of adventitious root (AR) determines the success of cutting-based propagation, and auxin is an essential factor involved in this process. To understand the molecular mechanism underlying AR formation in nodal tea cuttings, transcriptome and endogenous hormone analysis was performed on the stem bases of red (mature)- and green (immature)-stem cuttings of ‘Echa 1 hao’ tea plant as affected by a pulse treatment with naphthalene acetic acid (NAA).

**Results:**

In this study, NAA significantly promoted AR formation in both red- and green-stem cuttings but slightly reduced callus formation. External application of NAA reduced the levels of endogenous indole-3-acetic acid (IAA) and cytokinin (TZR, trans-zeatin riboside). The number of DEGs (NAA vs. CK) identified in the green-stem cuttings was significantly higher than that in the red-stem cuttings, which corresponded to a higher rooting rate of green-stem cuttings under the NAA treatment. A total of 82 common DEGs were identified as being hormone-related and involved in the auxin, cytokinin, abscisic acid, ethylene, salicylic acid, brassinosteroid, and jasmonic acid pathways. The negative regulation of NAA-induced *IAA* and *GH3* genes may explain the decrease of endogenous IAA. NAA reduced endogenous cytokinin levels and further downregulated the expression of cytokinin signalling-related genes. By the use of weighted gene co-expression network analysis (WGCNA), several hub genes, including three [cellulose synthase (*CSLD2*), SHAVEN3-like 1 (*SVL1*), SMALL AUXIN UP RNA (*SAUR21*)] that are highly related to root development in other crops, were identified that might play important roles in AR formation in tea cuttings.

**Conclusions:**

NAA promotes the formation of AR of tea cuttings in coordination with endogenous hormones. The most important endogenous AR inductor, IAA, was reduced in response to NAA. DEGs potentially involved in NAA-mediated AR formation of tea plant stem cuttings were identified via comparative transcriptome analysis. Several hub genes, such as *CSLD2*, *SVL1* and *SAUR21*, were identified that might play important roles in AR formation in tea cuttings.

**Supplementary Information:**

The online version contains supplementary material available at 10.1186/s12870-022-03701-x.

## Background

Tea plant [*Camellia sinensis* (L.) O. Kuntze], an important industrial crop species of the *Camellia* genus, originates from subtropical areas but is currently cultivated and consumed worldwide [1–3]. Tea is the most popular non-alcoholic beverage in the world and is produced from the young leaves of tea plant. Tea is popular not only because of its good taste and aroma but also because of its beneficial bioactive ingredients, such as tea polyphenols and theanine [4, 5].

Tea plant breeding occurs via both sexual reproduction and asexual reproduction. Sexual reproduction can result in increased numbers of mutations, recombination, and diversity and can possibly lead to new traits or beneficial qualities. However, sexual reproduction of tea plant also involves self-incompatibility and separation of individual progeny traits and can result in great differences in progeny. To maintain the characteristics of excellent varieties and facilitate rapid propagation, asexual propagation has been widely used in production [6, 7]. Propagation via cuttings is the main method of asexual propagation of tea plant and has been used for more than 200 years in China. Plants root systems are typically composed of primary root, lateral root (LR), and adventitious root (AR) [8]. Usually, the formation of AR determines the success of cutting-based propagation of tea plant, and rooting quality directly affects the survival rate of tea plant cuttings. The formation of AR involves a complex genetic process and is influenced by many internal and external factors, among which the phytohormone auxin plays a central role [8–10].

As an inducer of AR, auxin interacts with other plant hormones such as cytokinins, ethylene and jasmonates to jointly regulate the formation of AR [11]. Depending on the plant genotype and on other conditions, cuttings produce ARs independent or dependent on external auxin supply. In the latter case, cuttings are usually soaked in auxin before planting to promote AR formation which thus improves survival. Indole-3-acetic acid (IAA) is the most important physiologically active endogenous auxin in plants. However, due to the instability and easy degradation of IAA, naphthalene acetic acid (NAA) and indole-3-butyric acid (IBA) are commonly used to induce adventitious roots in practice [12]. As a synthetic auxin, NAA plays an important role in promoting cell division and expansion, inducing the formation of AR, increasing fruit setting, and preventing fruit dropping [13]. Compared with untreated ones, tea plant cuttings pretreated with NAA show better rooting activity and have a higher survival rate [9]. Studies have shown that, at low concentrations, NAA promotes the formation of AR of plants but inhibits rooting at high concentrations [14–17]. For example, compared with the control treatment, 200 mg/L NAA showed the best improvement in the rooting ability of *Hemarthria compressa*, while 400 mg/L NAA significantly reduced AR formation [17].

In previous studies, we investigated the effect of IBA treatment on the gene expression of tea plant stem cuttings by transcriptome sequencing and suppressive subtractive hybridization (SSH) [18, 19]. However, the mechanism through which NAA affects the rooting of tea plant cuttings is still unknown. Usually, each part of annual tea branches can be used for cuttings, but the upper green (immature) stem cuttings and the lower red (mature) stem cuttings have different rooting rates. The effects of NAA on AR formation, endogenous hormone levels and gene expression in tea plant cuttings at different maturity levels are worth further study.

In this study, we investigated the effects of NAA treatment on the callus induction rate, rooting rate, and death rate of red- and green-stem cuttings of tea plant. In addition, changes in the levels of five endogenous hormones in the stem bases of red- and green-stem cuttings were measured in response to NAA treatment. Furthermore, to explore the molecular mechanisms through which NAA promotes rooting of tea plant cuttings, transcriptome analysis was conducted on the stem bases of the red- and green-stem cuttings of ‘Echa 1 hao’ tea plant. These data will help to understand the mechanism of NAA’s involvement in promoting the rooting of tea plant cuttings.

## Results

### Effect of NAA treatment on callus formation and AR formation

**Fig. 1:**
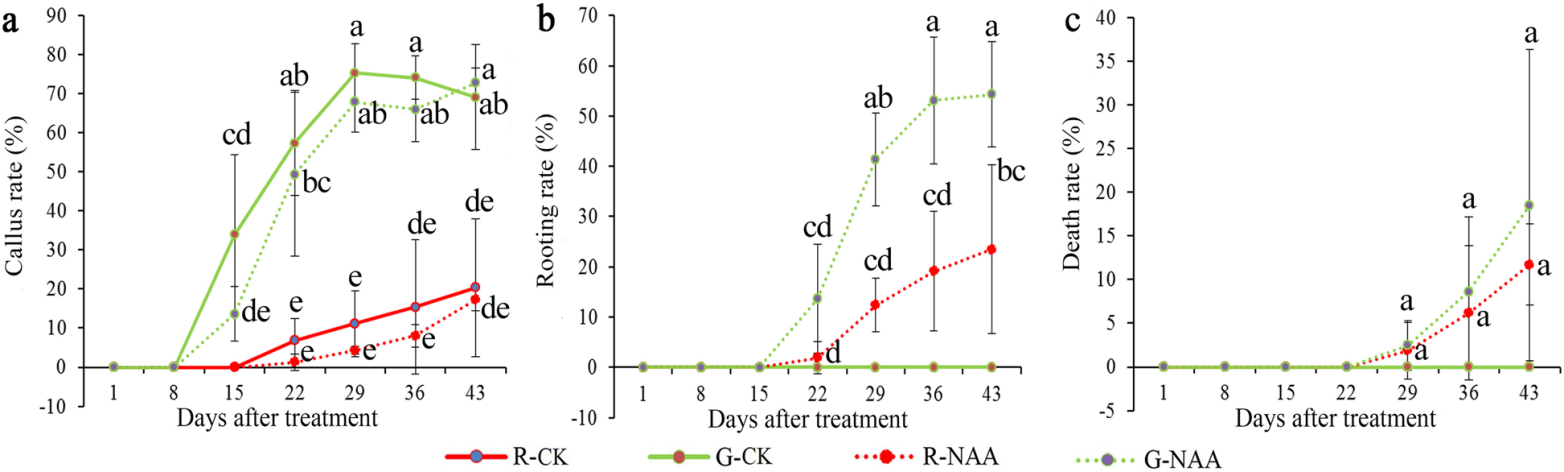
Effects of NAA treatment on AR formation of the red- and green-stem cuttings of tea plant. **a** Callus induction rate. **b** Rooting rate. **c** Death rate. Data represent the means ± SD of three independent replicates. Different lowercase letters indicate significant differences among all samples at *P* < 0.05. R-CK, Control group of red-stem cuttings; R-NAA, NAA treatment group of red-stem cuttings; G-CK, Control group of green-stem cuttings; G-NAA, NAA treatment group of green-stem cuttings

To study the effect of NAA on the rooting of tea plant cuttings with different maturity levels, red- and green-stem cuttings of tea plants were treated with 150 mg/L NAA or without NAA. The callus formation and AR formation status of the tea plant cuttings were investigated at 0, 1, 8, 15, 22, 29, 36, and 43 days (Fig. 1). In the absence of NAA, callus formation of green-stem cuttings occurred at 15 days, while that of red-stem cuttings occurred at 22 days (Fig. 1a). The callus induction rate of green-stem cuttings (highest 75.31%) was much higher than that of red cuttings (highest 20.37%). The callus induction rates of tea plant cuttings treated with NAA were transiently slightly lower than those of the control groups for both the green- and red-stem cuttings. NAA treatments promoted rooting of both green and red cuttings, while no roots were present in the control group even on day 43 (Fig. 1b). On day 22, rooting was observed for both green- and red-stem cuttings. As time progressed, the rooting rate of the cuttings increased gradually; the rooting rate of the green-stem cuttings was higher than those of the red-stem cuttings. No cuttings died under 150 mg/L NAA treatment, while both the green- and red-stem cuttings of the control groups began to die at 29 days, and the death rates of the green-stem cuttings were higher than those of the red-stem cuttings (Fig. 1c).

### Effect of NAA treatment on endogenous hormone contents

**Fig. 2:**
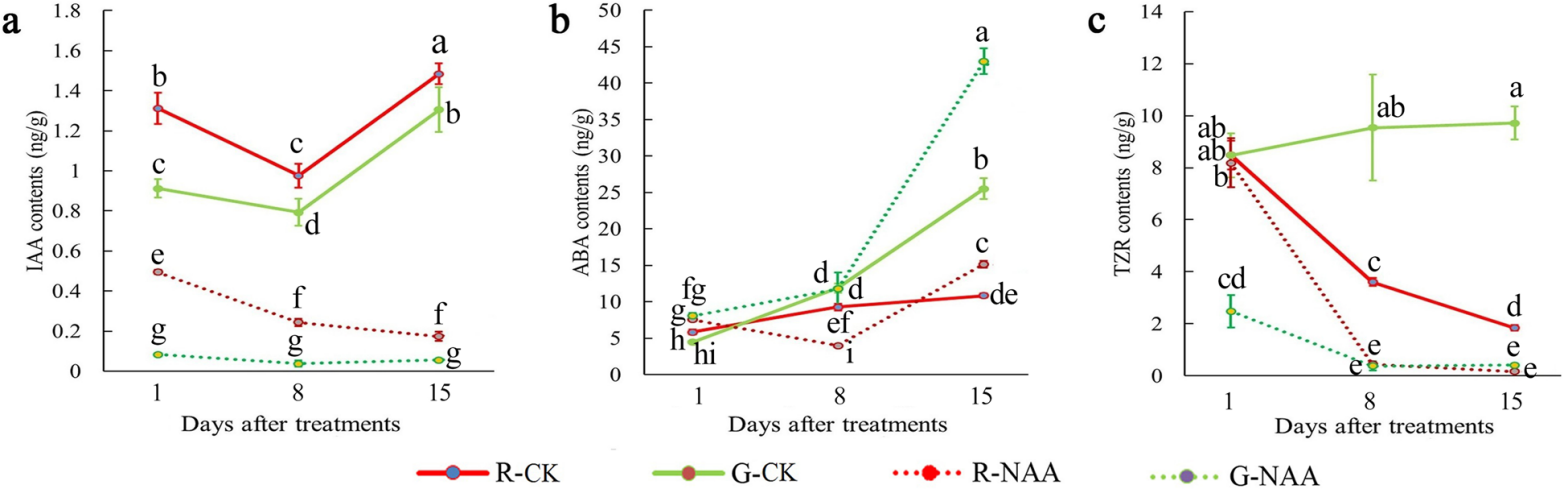
Effects of NAA treatment on endogenous hormone content changes in the base of red- and green-stem cuttings of tea plant. **a** IAA contents. **b** ABA contents. **c** TZR contents. Data represent the means ± SD of three independent replicates. Different lowercase letters indicate significant differences among all samples at *P* < 0.05. R-CK, control group of red-stem cuttings; R-NAA, NAA treatment group of red-stem cuttings; G-CK, control group of green-stem cuttings; G-NAA, NAA treatment group of green-stem cuttings

To investigate the effect of NAA treatment on endogenous hormones, stem bases of cuttings were collected on days 1, 8, and 15 for the determination of endogenous hormone contents, including IAA, abscisic acid (ABA), gibberellic acid 1 (GA1), gibberellic acid 3 (GA3), and trans-zeatin riboside (TZR) (Fig. 2 and Fig. S1). The results showed that NAA treatment significantly reduced endogenous IAA contents in the bases of both the red- and green-stem cuttings (Fig. 2a). The content of IAA in the green-stem cuttings was lower than that in red-stem cuttings. In the early stage, the ABA contents of the red and green-stem cuttings were similar (Fig. 2b). However, as time progressed, the ABA content of the green-stem cuttings became higher than that of the red-stem cuttings; at day 15, stem bases of NAA-treated cuttings had higher ABA levels when compared to control cuttings. TZR decreased significantly in both red- and green-stem cuttings after NAA treatment (Fig. 2c). The contents of GA1 and GA3 were more variable. In the NAA-treated groups, GA1 showed a trend of decreasing first and then increasing in the green-stem cutting group, while it was the opposite for the red-stem cuttings (Supplementary Fig. S1). In the control group, the green stems had a higher GA3 content than the red stems did. NAA treatment decreased the GA3 content in the green-stem cuttings but increased the GA3 content in the red-stem cuttings.

### Transcriptome assembly and DEG analysis

The Q30 percentage was > 86.19% in each library, with an average of 91.11%, indicating that the clean data were of high quality and could be used for subsequent analysis. More than 85% (85.36%–90.69%) of the clean reads in each sample mapped to the tea plant reference genome (Table 1). The correlation of gene expression levels showed that all biological replicates of the samples were highly correlated (Supplementary Fig. S2).

**Table 1:** Quality of the transcriptome of tea plant cuttings

Sample	Sample codes	Total raw reads	Total clean reads	Q30 percentage	GC percentage	Mapped reads
R-CK-1d	A1	55,748,520	54,902,578	90.89%	44.68%	48,461,126 (88.27%)
	A2	45,180,146	44,519,734	90.79%	44.67%	39,218,588 (88.09%)
	A3	56,566,114	55,926,244	91.78%	44.68%	49,444,924 (88.41%)
G-CK-1d	B1	44,381,646	43,750,294	92.05%	44.45%	39,676,319 (90.69%)
	B2	52,844,132	52,205,426	91.42%	44.45%	47,188,315 (90.39%)
	B3	52,031,904	51,009,182	91.94%	44.42%	46,167,529 (90.51%)
R-NAA-1d	C1	57,542,312	56,686,212	91.89%	44.59%	51,029,371 (90.02%)
	C2	55,347,558	54,541,818	91.59%	44.45%	48,816,430 (89.5%)
	C3	51,652,776	51,038,136	91.47%	44.55%	45,737,006 (89.61%)
G-NAA-1d	D1	52,700,594	52,057,588	90.97%	44.23%	46,509,280 (89.34%)
	D2	55,390,048	54,436,326	90.91%	44.31%	48,726,670 (89.51%)
	D3	56,837,616	55,965,548	91.10%	44.25%	50,099,962 (89.52%)
R-CK-8d	E1	46,872,348	46,240,196	92.72%	44.68%	41,131,620 (88.95%)
	E2	49,103,480	48,415,422	91.14%	44.67%	42,851,995 (88.51%)
	E3	50,104,932	49,475,110	91.93%	44.70%	44,022,857 (88.98%)
G-CK-8d	F1	63,410,740	62,124,940	90.87%	44.42%	55,965,379 (90.09%)
	F2	50,629,854	49,770,586	90.94%	44.59%	45,090,269 (90.6%)
	F3	54,849,538	53,952,010	91.47%	44.34%	48,785,963 (90.42%)
R-NAA-8d	G1	67,137,970	66,312,940	90.96%	44.62%	59,403,835 (89.58%)
	G2	53,263,604	52,548,220	89.24%	44.64%	46,583,734 (88.65%)
	G3	63,705,624	62,386,776	91.73%	44.61%	55,864,525 (89.55%)
G-NAA-8d	H1	55,555,552	54,971,598	91.33%	44.68%	49,699,991 (90.41%)
	H2	40,449,238	39,861,204	86.19%	44.33%	34,024,657 (85.36%)
	H3	48,181,458	47,547,582	88.58%	44.38%	41,066,507 (86.37%)
R-CK-15d	I1	47,395,304	46,844,706	90.23%	44.46%	41,872,539 (89.39%)
	I2	54,717,128	54,094,692	90.30%	44.48%	48,221,365 (89.14%)
	I3	60,353,348	59,666,972	89.08%	44.48%	52,824,484 (88.53%)
G-CK-15d	J1	41,621,104	41,221,466	91.20%	44.49%	37,187,978 (90.22%)
	J2	54,133,590	53,526,422	91.27%	44.73%	48,540,696 (90.69%)
	J3	60,629,848	59,836,052	90.28%	44.42%	53,573,924 (89.53%)
R-NAA-15d	K1	46,668,718	45,880,264	92.31%	44.63%	41,307,179 (90.03%)
	K2	68,372,780	66,772,728	92.20%	44.56%	60,021,696 (89.89%)
	K3	55,461,778	54,518,126	92.59%	44.67%	49,094,849 (90.05%)
G-NAA-15d	L1	47,265,242	46,458,626	92.44%	44.69%	41,911,565 (90.21%)
	L2	48,935,976	48,099,790	91.55%	44.62%	43,335,448 (90.09%)
	L3	48,331,024	47,521,016	92.14%	44.73%	43,008,727 (90.5%)

Samples A, E, I represent red-stem cuttings (1, 8 and 15 days) under control. Samples B, F, J represent green-stem cuttings (1, 8 and 15 days) under control. Samples C, R, K represent red-stem cuttings (1, 8 and 15 days) under NAA treatment. Samples D, H, L represent green-stem cuttings (1, 8 and 15 days) under NAA treatment

**Fig. 3:**
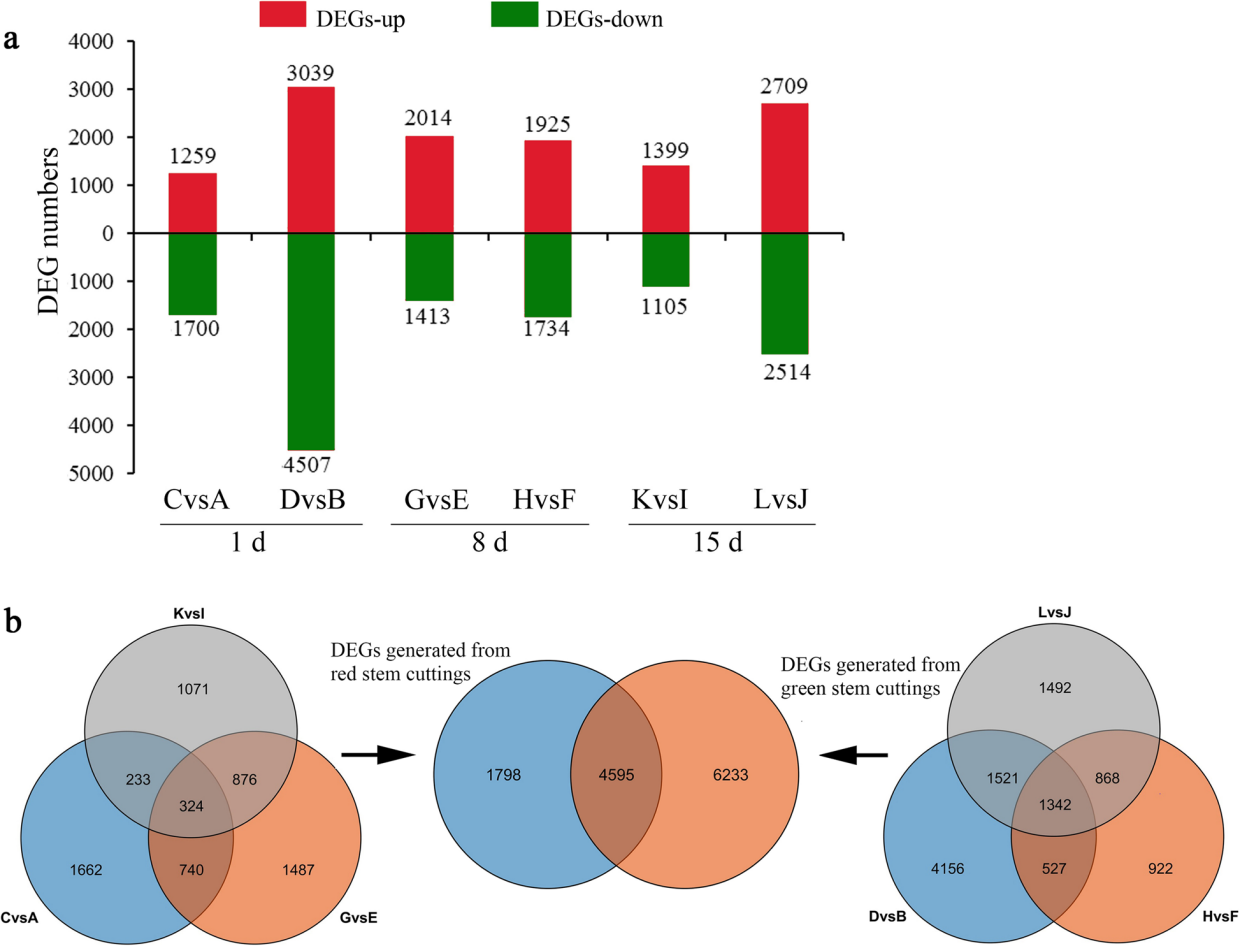
DEGs (NAA vs. CK) identified in different comparisons under NAA treatment. **a** Number of DEGs identified in different comparisons. **b** Venn diagram of DEGs between the red- and green-stem cutting groups. C vs A, D vs B and G vs E represented DEGs of red-stem cutting at 1, 8 and 15 days, respectively; H vs F, K vs I and L vs J represented DEGs of green-stem cutting at 1, 8 and 15 days, respectively

When the NAA-treated and control tea plant cuttings were compared, 2959 (C vs A), 3427 (G vs E) and 2504 (K vs I) DEGs (differentially expressed genes) were identified in the red-stem cuttings, and 7546 (D vs B), 3659 (H vs F) and 5223 (L vs J) were identified in green-stem cuttings on days 1, 8 and 15, respectively (Fig. 3a). The number of DEGs in green-stem cuttings was higher than that in the red-stem cuttings at all three time points, and the number of DEGs in the green-stem cuttings was 2.6, 1.1, and 2.1 times than that in the red-stem cuttings on days 1, 8, and 15, respectively. The number of DEGs in the green stems decreased first and then increased, while that in the red stems increased first and then decreased. Both red- and green-stem cuttings had more downregulated genes on day 1, and more upregulated genes on day 8 and 15. To discover the common transcriptional changes under NAA treatment, we compared the DEGs involved in the green- and red-stem cuttings and identified 4595 DEGs belonging to the overlapp of 6393 DEGs from the red-stem cuttings and 10,828 DEGs from the green-stem cuttings (Fig. 3b). The 4595 DEGs common to both the red- and green-stem cutting groups were used for further analysis.

### Gene ontology and KEGG enrichment analysis of DEGs

To determine the major responsive mechanism underlying AR formation, Gene Ontology (GO) and KEGG pathway enrichment analyses were performed on the 6393 DEGs from the red-stem cuttings, 10,828 DEGs from the green-stem cuttings, and 4595 DEGs common to both the red- and green-stem cuttings.

**Fig. 4:**
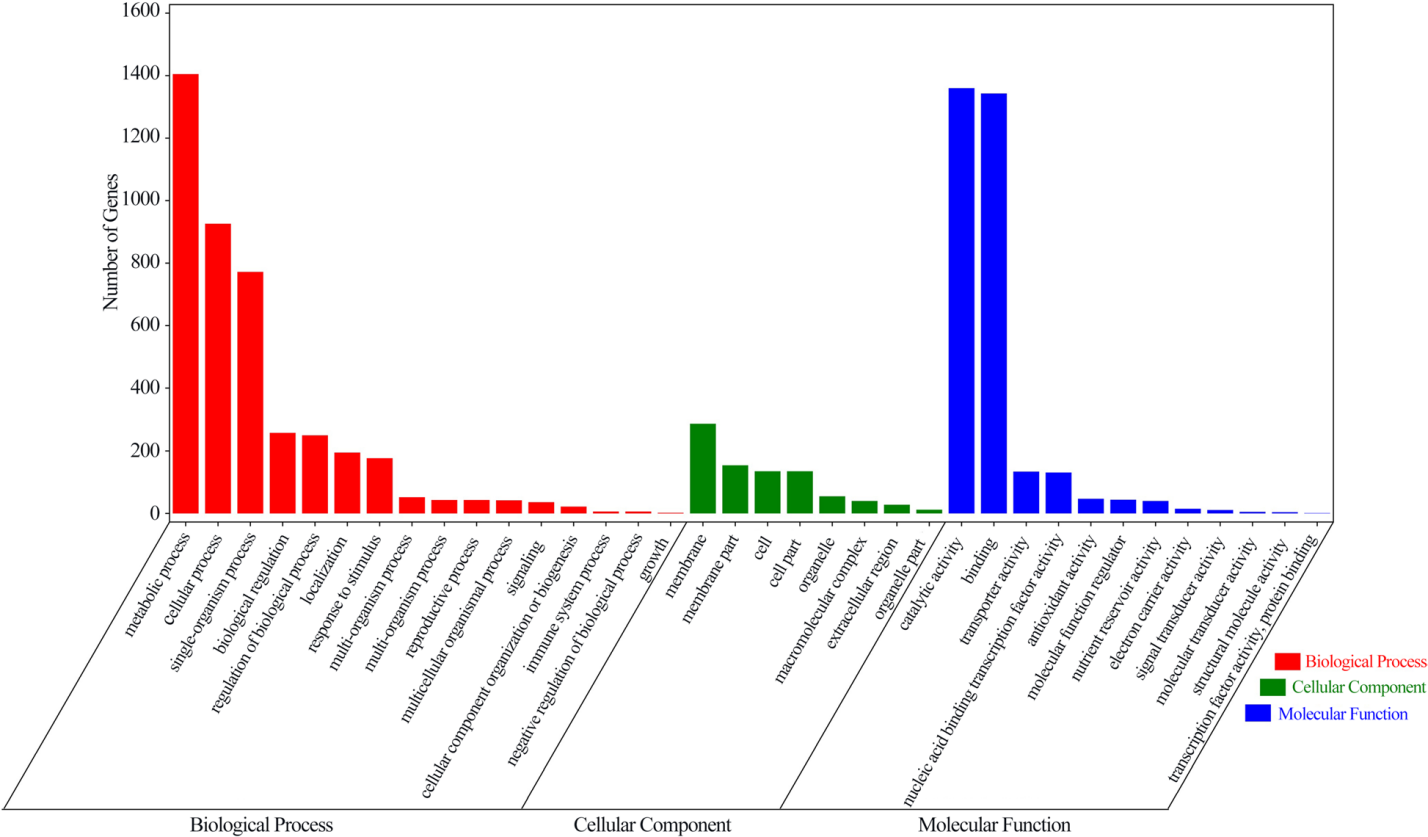
GO enrichment analysis of DEGs (NAA vs. CK) common to both red- and green-stem cutting groups

As shown in Fig. 4 and Supplementary Fig. S3, most of the GO terms were similar in the three sets. According to the GO annotations, unigenes were classified into different functional categories. The terms ‘metabolic process’ and ‘cellular process’ in the ‘biological process’ category contained the most DEGs. For the ‘molecular function’ category, the most DEGs were associated with ‘binding’ and ‘catalytic activity’. For the ‘cellular component’ category, the most DEGs were associated with ‘membrane’.

**Fig. 5:**
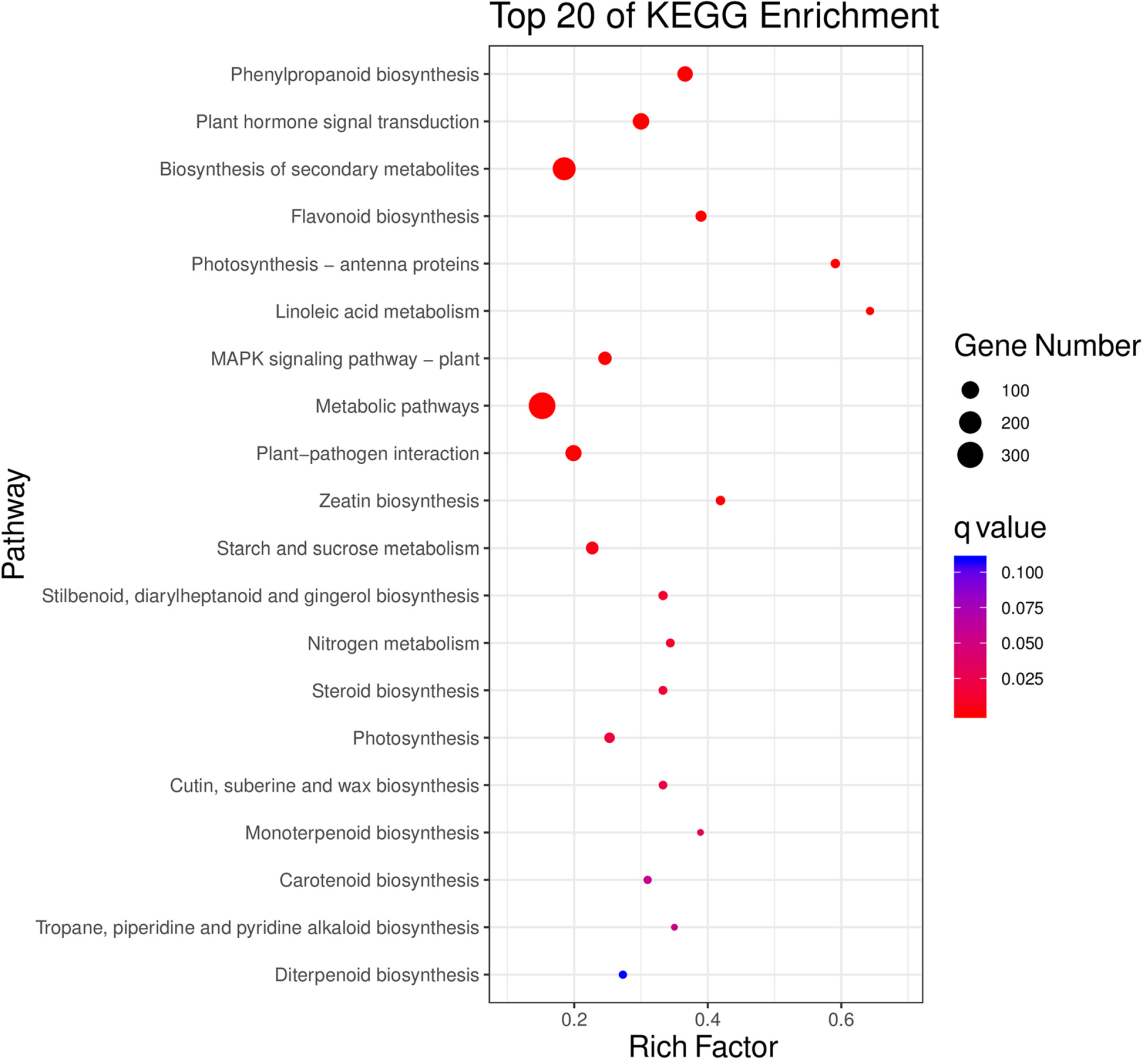
KEGG enrichment analysis of DEGs (NAA vs. CK) common to both red- and green-stem cutting groups. The ‘Rich Factor’ means the number of genes belonging to that pathway in the target gene set/the number of all the genes in that pathway in the background gene set. Permissions to use the KEGG pathway map was taken from the Kanehisa Laboratories (https://www.kanehisa.jp/)

KEGG analysis was performed on all identified DEGs to analyse the canonical pathways. A total of 687 DEGs were annotated and assigned to 112 pathways out of the 4595 DEGs shared by the red- and green-stem cuttings (Supplementary Table S1). In addition, a total of 1431 and 853 DEGs were annotated and assigned to 126 and 117 pathways from the green- and red-stem cutting groups, respectively (Supplementary Tables S2 and S3). The top 20 KEGG pathways were determined according to a *p*-adjusted value < 0.05 (Fig. 5 and Supplementary Fig. S4). Twelve pathways, i.e., ‘phenylpropanoid biosynthesis’, ‘plant hormone signal transduction’, ‘biosynthesis of secondary metabolites’, ‘plant-pathogen interaction’, ‘metabolic pathways’, ‘MAPK signalling pathway-plant’, ‘zeatin biosynthesis’, ‘photosynthesis-antenna proteins’, ‘flavonoid biosynthesis’, ‘starch and sucrose metabolism’, ‘nitrogen metabolism’, and ‘monoterpenoid biosynthesis’, were consistent among the three sets. Among them, ‘phenylpropanoid biosynthesis’ and ‘plant hormone signal transduction’ were the most representative pathways.

### DEGs involved in plant hormone signal transduction pathways

**Fig. 6:**
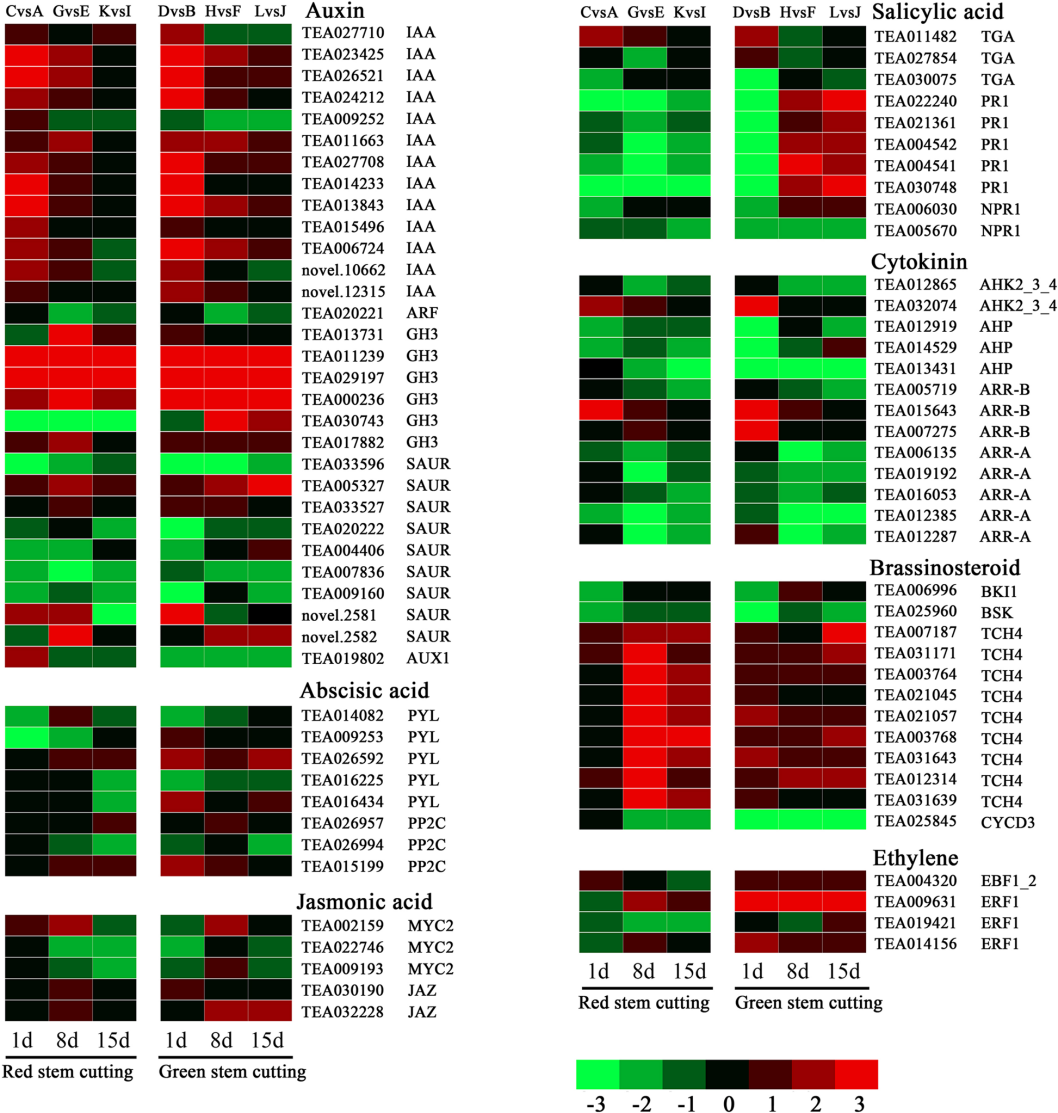
Expression analysis of common DEGs (NAA vs. CK) involved in plant hormone signal transduction pathways. The different colours indicate DEG expression levels based on the log_2_(fold change) values. The green module represents down regulated expression, and the red module represents up regulated expression

A total of 82 common DEGs were identified as being hormone related, which included DEGs involved in the auxin, cytokinin, ABA, ethylene, salicylic acid, brassinosteroid, and jasmonic acid pathways (Fig. 6 and Supplementary Table S4). Most of the DEGs showed the same upregulated or downregulated expression tendency in both the red- and green-stem cutting groups. However, several salicylic acid-related genes, *PR1* (pathogenesis-related protein 1), TEA022240, TEA021361, TEA004542, TEA004541, and TEA030748, were downregulated in the red-stem cuttings and upregulated in green-stem cuttings on days 8 and 15. A total of 30 DEGs were involved in the auxin pathway, which accounted for the largest proportion among all hormone-related DEGs. The auxin-related DEGs involved three major groups, *IAA* (auxin-responsive protein IAA), *GH3* (auxin-responsive GH3 gene family) and *SAUR* (SAUR family protein) proteins. Among them, the *IAA* and *GH3* DEGs mainly showed an increasing expression trend, while the *SAUR* DEGs mainly showed a decreasing expression trend. With NAA treatment, most cytokinin-related DEGs in both red- and green-stem cuttings were downregulated. In contrast, most brassinosteroid-related genes were upregulated. Several DEGs have also been identified as being involved in the ABA, jasmonic acid, and ethylene pathways. Ethylene-related genes showed a higher upregulated expression tendency in the green-stem cuttings than in the red-stem cuttings.

### Co-expression network analysis via WGCNA

**Fig. 7:**
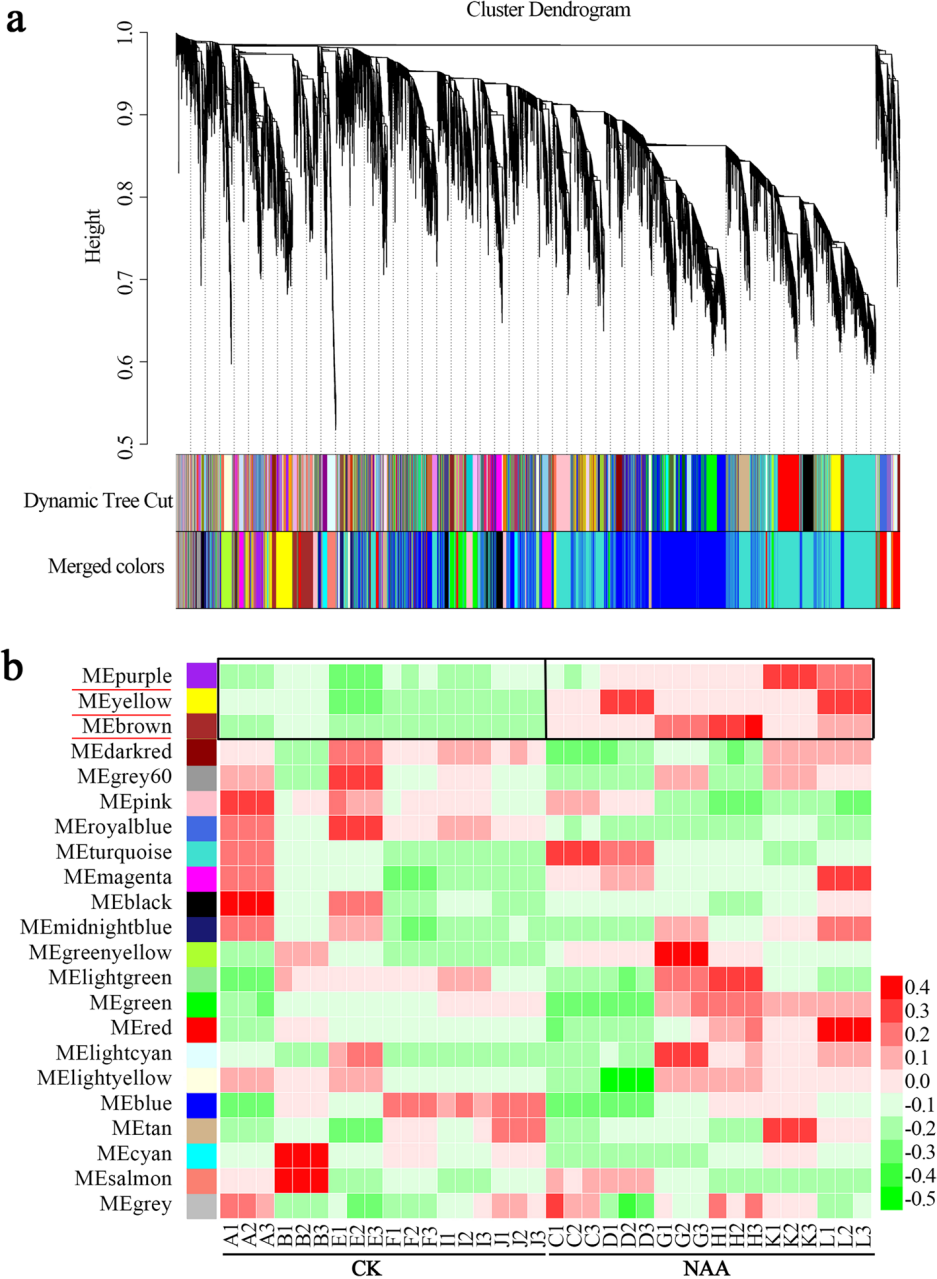
WGCNA of genes in the red- and green-stem cuttings that responded to NAA treatment. **a** Hierarchical cluster tree showing co-expression modules. Each leaf of the tree represents one gene. The major tree branches constitute 22 modules labelled by different colours. **b** Module–sample association. Each row corresponds to a module, and each column corresponds to a specific sample. The colour of each cell at the row-column intersection represents the correlation coefficient between the module and the sample. A high degree of correlation between a specific module and the sample is indicated by an underlined module name

To reveal the regulatory network correlated with NAA treatment across red- and green-stem cuttings, gene co-expression analysis was performed via weighted gene co-expression network analysis (WGCNA). In WGCNA, modules are defined as clusters of highly interconnected genes, and genes in the same cluster have a high correlation coefficient. A total of 22 distinct modules were identified (labelled with different colours) (Fig. 7a). Module–sample correlation analysis was then performed to determine the specific module that was highly correlated with the response to NAA treatment. Notably, three co-expression modules (purple, yellow and brown) were detected, which are underlined in red in Fig. 7b.

**Fig. 8:**
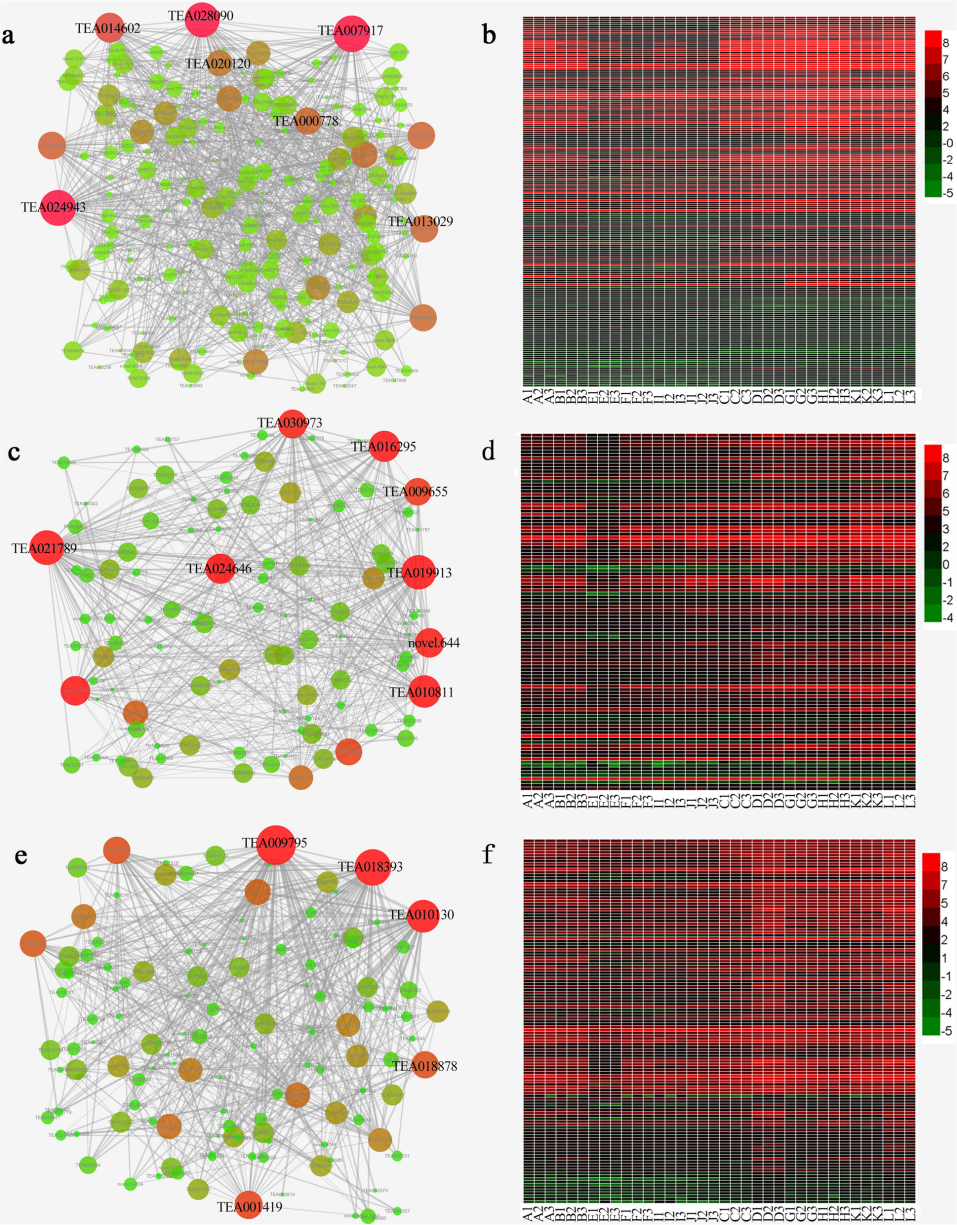
Co-expression network analysis of NAA-specific modules. **a**, **c**, **e** Gene regulatory network of the brown, purple and yellow modules, respectively. **b**, **d**, **f** Heatmaps of genes corresponding to the brown, purple and yellow modules respectively. Candidate hub genes are shown in red. samples A, E, I represent red-stem cuttings (1, 8 and 15 days) under control. Samples B, F, J represent green-stem cuttings (1, 8 and 15 days) under control. Samples C, R, K represent red-stem cuttings (1, 8 and 15 days) under NAA treatment. Samples D, H, L represent green-stem cuttings (1, 8 and 15 days) under NAA treatment

In the brown module, 1179 genes were identified specifically at 8 days of NAA treatment. We selected the coefficient relationships of the top 500 weights to construct the networks and determine the hub genes (Fig. 8a); hub genes are those that show the most connections in a network, as indicated by their high degree. As shown in Fig. 8b, the expression of the brown module genes was upregulated by NAA, especially at 8 days. The Helix-loop-helix DNA-binding Domain (bHLH) transcription factor was identified as the hub gene with the highest degree. Genes encoding the COBRA-like protein, Endoplasmic Reticulum Oxidoreductin 1 (ERO1), Uncharacterized protein At2g33490, UDP-glucose/GDP-mannose dehydrogenase family proteins, C3HC4-type (RING finger) proteins, and the SHAVEN3-like 1 (SVL1) protein were also identified as hub genes for this module (Table 2).

**Table 2:** Candidate hub genes in brown, purple, and yellow modules

Gene name	Degree	Description
**Brown module**		
TEA007917	49	Helix-loop-helix DNA-binding domain-containing protein
TEA028090	41	COBRA-like protein
TEA024943	38	Endoplasmic Reticulum Oxidoreductin 1 (ERO1)
TEA014602	33	Uncharacterized protein At2g33490
TEA000778	25	Zinc finger, C3HC4 type (RING finger)-protein
TEA013029	24	UDP-glucose/GDP-mannose dehydrogenase family protein NAD-binding domain-containing protein
TEA020120	23	SHAVEN3-like 1
**Purple module**		
TEA021789	56	RCD1-SRO-TAF4 (RST) plant domain-containing protein
TEA019913	55	Serine/threonine-protein kinase 11-interacting protein
TEA010811	49	AP2 domain-containg prograin
TEA016295	45	Glycosyl hydrolases family 17 protein
TEA024646	42	Cysteine-rich receptor-like protein kinase 3
novel.644	39	Protein kinase domain-containing protein
TEA030973	38	Cellulose synthase
TEA009655	34	G-box binding protein MFMR
**Yellow module**		
TEA009795	66	Caspase domain-containing protein
TEA018393	57	PAP2 superfamily C-terminalprotein
TEA010130	46	U-box domain/SMALL AUXIN UP RNA
TEA001419	33	KH domain-containing protein
TEA018878	30	GDP-mannose 4,6-dehydratase

The purple module genes were more highly expressed under NAA treatment for 15 days (Fig. 8d). The RCD1-SRO-TAF4 plant domain (RST), Serine/threonine-protein kinase 11-interacting protein, AP2 domain (AP2)-containing protein, Glycosyl hydrolase family 17 protein, Cysteine-rich receptor-like protein kinase 3 (CRK3), Protein kinase domain-containing proteins, Cellulose synthase (CSLD2), and the G-box binding protein MFMR were also discovered to be hub genes (Fig. 8c and Table 2).

The yellow module genes were more highly expressed in the NAA treatment at 1 and 15 days in green-stem cuttings (Fig. 8f). A co-expression network was constructed by the top 500 coefficient relationships of 117 genes (Fig. 8e). The co-expression results showed that the genes encoding the caspase domain-containing (MC1) protein, PAP2 superfamily C-terminal protein, U-box domain/SMALL AUXIN UP RNA (SAUR21), the KH domain-containing protein (HEN4), GDP-mannose 4,6-dehydratase, and oxysterol-binding protein were identified as hub genes in this network (Table 2).

### Verification of DEGs from RNA-seq by RT-qPCR

**Fig. 9:**
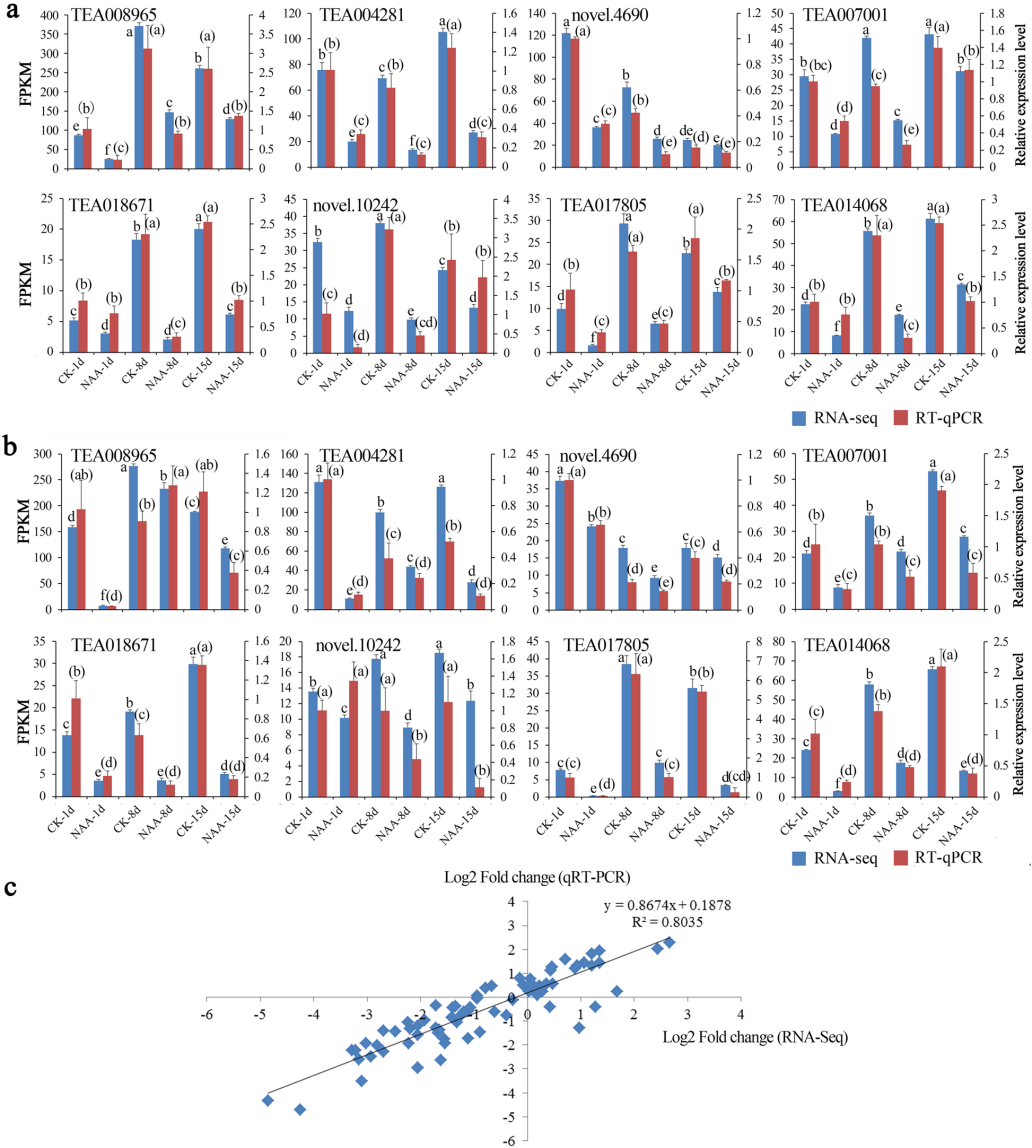
RT-qPCR-based verification of the RNA-seq analysis results. **a** Red-stem cutting groups. **b** Green-stem cutting groups. **c** Correlations of the expression levels of the DEGs measured via RNA-seq and RT-qPCR. The lowercase letters without parentheses present significant differences of RNA-seq data, and the lowercase letters in parentheses present significant differences of RT-qPCR data

To verify the reliability of gene expression from RNA-seq data, eight DEGs were randomly selected for RT-qPCR validation (Fig. 9). Compared with the RNA-seq analysis results, the results showed that most of the DEGs showed similar expression trends (Fig. 9a and b). In addition, correlation analysis showed that the RT-qPCR results were highly consistent (R^2^ = 0.8) with the RNA-seq results (Fig. 9c). Therefore, the RNA-seq results were reliable.

## Discussion

Currently, commercial tea plant cultivars are mainly propagated from stem cuttings that are separated from their mother plant, which ensures the stability of the cultivars. The induction and growth of AR is a key step in the vegetative propagation of plants, and new roots can either form spontaneously or be induced by certain stimuli from stem cuttings or other vegetative tissues [12, 20]. Several studies have shown that NAA induces adventitious root formation [17, 21]. However, few studies have reported the mechanism underlying the effect of NAA on tea plant cuttings with different maturity levels.

In this study, the effect of NAA as pulse treatment on AR induction of tea plant cuttings was analyzed. In both the absence and presence of NAA treatment, the cuttings still had a relatively high callus induction rate (especially in the case of the green-stem cuttings), but no rooting occurred in the absence of NAA, indicating that callus formation and rooting are two independent processes and that there is no direct relationship between them. NAA treatment significantly promoted the rooting rate but no significant differences were observed for callus induction rate. The callus induction rate and rooting rate of the green-stem cuttings were significantly higher than those of the red-stem cuttings, which is consistent with the findings of Yan et al. [17], who reported that the stem cuttings of *Hemarthria compressa* obtained in 2012 had better rooting characteristics than did those obtained in 2011. AR formation is a multiphase process, and the most widely recognized AR phases are induction, initiation and expression, in which the initial induction phase has contrasting hormonal requirements compared to the subsequent initiation and expression phases [11, 22]. The induction phase is usually the first few hours after cutting removal, with no visible cell division, involving reprogramming of target cells [11]. During the initial stage of AR, the cell structure undergoes qualitative changes, and the cells differentiate into root primordia. The final expression phase begins with the differentiation of primordia into the complete root body, follows by the emergence of roots. In this study, although first roots were emerged from the stem at day 22, the AR induction of tea cuttings must have occurred earlier; day 1 was probably the very early induction phase, and days 8 and 15 may have covered the induction and initiation/expression phases, respectively.

Auxin normally coordinates with other hormone signals to regulate cellular processes such as division, elongation and differentiation [23]. Exogenous auxin affects the balance of endogenous hormones, resulting in changes in plant growth and development. Many studies have shown that exogenous auxin promotes rooting by increasing endogenous IAA contents [8, 24]. For example, NAA-treated soybean hypocotyl cuttings showed a significant increase in the number of AR, an increase in endogenous IAA, and a decrease in IAA-oxidase (IAAO) activity [25]. NAA treatment at 500 mg/L had the best rooting effect on cuttings of *Carya illinoinensis*, and NAA significantly increased the endogenous IAA content in the early stages [21]. However, Ribnicky et al. reported that NAA mainly was present in a conjugated form and had little influence on the concentration of endogenous IAA [26]. The present study indicated that NAA reduced the content of endogenous IAA contents in both red- and green-stem cuttings. Similarly, Chen et al. reported that NAA reduced endogenous IAA contents in cuttings of *Populus tomentosa* and *Populus davidiana*, which is consistent with our research [27]. This study suggests that NAA may directly stimulate cell division and differentiation in the cambial region of tea plant cuttings, thereby promoting AR formation rather than acting by increasing endogenous IAA biosynthesis; the decrease in endogenous IAA content may be due to NAA binding to auxin transporters and thus reducing the amount of IAA generated in the shoot tips from reaching the bottom of the stems.

Exogenous NAA also alters the levels of other endogenous hormones. ABA is generally considered a rooting inhibitor of cuttings [22, 28]. However, this study showed that NAA slightly increased ABA levels and that ABA content increased over time, especially in the green-stem cuttings. The results suggest that ABA is not a key hormone needed for rooting; however, ABA, an important plant stress hormone, improves the stress resistance of cuttings by promoting excessive catabolism of stored nutrients [29, 30]. Here, the changes in ABA content are more likely a synergistic response to the changes of other hormones. Auxin and cytokinin usually have antagonistic effects in adventitious root formation [31, 32]. A high auxin:cytokinin ratio promotes the formation of AR, while a relatively low ratio facilitates shoot formation. A recent study of *Rosa* hybrida showed that the better rooting of higher position cuttings was based on a higher IAA level and IAA/cytokinin ratio in the stem base, and the outgrowing axillary bud obviously contributed to the IAA accumulation in the stem base [33]. In this study, exogenous NAA significantly reduced the content of TZR (the most active cytokinin) in the tea plant cuttings. This is consistent with the conclusion that relatively low amounts of cytokinins promote root formation. The TZR of untreated red-stem cuttings also decreased over time. The underlying mechanism still needs further study. Compared with other hormones, the contents of GA1 and GA3 were more variable, suggesting that they might not be stable in cuttings (Supplementary Fig. 1).

To better understand the AR formation mechanism of tea plant cuttings, transcriptome analysis was conducted on the stem bases of red- and green-stem cuttings treated with or without NAA. The number of DEGs identified in the green-stem cuttings was significantly higher than that in the red-stem cuttings, which was consistent with the fact that the green-stem cuttings showed a higher rooting rate after NAA treatment. Among the hormone-related DEGs, the auxin pathway accounted for the largest proportion, which also reflects the critical role of auxin in AR formation in tea plant cuttings. *IAA* and *GH3* are auxin-responsive genes that have negative regulatory feedback effects on the auxin response [34, 35]. GH3 enzymes regulate auxin concentrations by promoting the conjugation of amino acids and IAA [36]. In addition, GH3 proteins function as acyl acid amido synthetases, which can also conjugate jasmonic acid or SA depending on the specific gene [37, 38]. Here, the expression of three *GH3* (TEA011239, TEA029197, TEA000236) genes was induced in response to NAA during the rooting process of tea plant cuttings, among which the highest expression levels of TEA011239 and TEA029197 were upregulated by 707.5- and 59.3-folds, respectively. Similarly, our previous study also revealed that two *GH3* genes were highly induced by IBA [19]. The negative regulation of NAA-induced *IAA* and *GH3* genes may explain the decrease of endogenous IAA. Several SMALL AUXIN UP RNAs (SAURs) have been identified as being involved in auxin-induced expression in different plant species [39, 40]. Dark incubation enhanced the accumulation of IAA in the stem base during AR induction of petunia cuttings, which was related to up- versus down-regulation of distinct *SAUR* genes [40]. In shoots and primary roots, specific SAURs act dependent on the TIR1-Aux/IAA machinery and control cell expansion via targeting PP2C. D phosphatases [40].

A previous study showed that IBA could inhibit the expression of cytokinin synthesis-related genes such as isopentyltransferase adenylate and cytokinin hydroxylase and induce the expression of cytokinin degradation-related genes [19]. In this study, exogenous NAA inhibited the expression of most DEGs involved in the cytokinin signalling pathway, which was consistent with the decrease in endogenous cytokinin content after NAA treatment. A histidine phosphate transfer protein (TEA013431) gene was specifically identified. Its expression level under control reached 192-folds higher than that under NAA treatment. In *Arabidopsis thaliana*, histidine phosphate transfer proteins (AHPs) function as positive regulators of cytokinin signalling, which activates *A. thaliana* response regulators (ARRs) by phosphorylation [41]. Taken together, these results suggest that NAA reduces endogenous cytokinin and thus downregulates the expression of cytokinin signalling-related genes.

Brassinosteroids (BRs) may also be important plant hormones involved in AR formation. When plants are treated with exogenous auxin, BRs have been shown to play a synergistic role in promoting cell elongation [42]. TCH4 belongs to the xyloglucan endotransglycosylase gene family (XET). In *A. thaliana*, BRs regulate cell wall elongation by increasing *XET* expression levels, and the expression levels of *TCH4* and *AtXTH24* are greatly downregulated in *dwf1* (a BR synthesis mutant) [43]. In the present study, all nine *TCH4* (TEA007187, TEA031171, TEA003764, TEA021045, TEA021057, TEA003768, TEA031643, TEA012314, and TEA031639) genes were upregulated in tea plant, suggesting that auxin may promote AR formation by increasing BR contents.

Ethylene has a positive effect on AR formation and is closely related to the activity of auxin [44]. The expression of many ethylene responsive transcription factors (ERFs) is induced during AR formation and in injured leaves [44]. Here, three ERF genes were identified, among which both TEA009631 and TEA014156 were induced at different stages of green-stem cuttings, while downregulated expression was observed in the red-stem cuttings. This may be the reason for the difference in rooting of the cuttings from different aged stems.

ABA, jasmonic acid, and salicylic acid are stress hormones [45], and the expression of their related genes was observed to be altered by NAA treatment. These hormones may also be involved in AR formation. The induction of most hormone-related DEGs in the green stems was higher than that in the red-stems during rooting. Notably, several salicylic acid-related genes, *PR1*, TEA022240, TEA021361, TEA004542, TEA004541, and TEA030748, were downregulated throughout the rooting process in the red-stem cuttings but upregulated at the later stage in the green-stem cuttings. The role of jasmonic acid in AR formation is much more complex [11]. Jasmonic acid-deficient *A. thaliana* mutants had higher rooting ability than did the wild-type plants [46, 47]. In *Arabidopsis*, interactions of three specific auxin-induced *GH3* genes control the reduction of jasmonic acid levels by catalyzing the conjugation of jasmonic acid to amino acids during AR initiation, thus promoting the formation of AR in etiolated seedlings [47]. However, jasmonic acid -deficient cuttings of *Petunia* hybrida showed reduced AR formation [11, 48].

WGCNA provides a useful approach for identifying sample-specific modules and candidate hub genes. In soybean, the regulatory networks and hub genes controlling seed set and size were identified via WGCNA [49]. Recently, WGCNA was also used to identify key hub genes in response to nitrogen treatment of tea plant [50]. However, the application of co-expression analysis during rooting to identify hub genes has not been reported. In this study, WGCNA was first applied in rooting promotion of NAA. A total of 22 co-expression modules were constructed, including 3 modules that were significantly related to NAA treatment. As a hub gene, *SVL1* (TEA020120) was identified in the brown module in this study, and this gene was shown to be involved in cell wall cellulose accumulation and pectin linkage and affected the development of root hair, trichoid and epidermal cells [51]. *CSLD2* (TEA030973) was identified in the purple module as a cellulose synthase protein required in the late development of root hairs; this protein polymerizes the skeleton of non-cellulose polysaccharides (hemicelluloses) in plant cell walls [52]. The *SMALL AUXIN UP RNA* (*SAUR*) gene is a member of the largest family of auxin response genes. Studies have shown that the expression of the *SAUR* gene may be related to auxin-mediated cell expansion, but the function of this gene is still unclear [53]. As a *SAUR* gene, *SAUR21* (TEA010130) was also identified as a hub gene in the yellow module and was highly related to NAA treatment. The discovery of hub genes provides target genes for further research and also plays an important role in the breeding of improved varieties in the future.

## Conclusions

The present study revealed that AR formation in both red- and green-stem cuttings of tea plants was dependent on a pulse treatment with NAA, while green-stem cuttings showed a better rooting. Exogenous NAA significantly reduced the contents of endogenous IAA and cytokinin TZR. The number of DEGs (NAA vs. CK) identified in the green-stem cuttings was significantly higher than that in the red-stem cuttings, which corresponded to a higher rooting rate of green-stem cuttings under the NAA treatment. DEG enrichment analysis also confirmed that many key genes (such as, *IAA* and *GH3*, etc.) involved in plant hormone signal transduction were affected by NAA and significantly changed during AR formation process of tea plants. Our study will be helpful to further understand the AR formation mechanism of tea plant cuttings.

## Methods

### Plant materials and treatments

Both red- (mature) and green- (immature) stem nodal cuttings were collected from *C. sinensis* (L.) O. Kuntze *cv.* ‘Echa 1 hao’. The standard length of each cutting was approximately 3 cm, each with a mature leaf and a plump axillary bud. For disinfection, the cuttings were immersed in 1% carbendazim for 5 min and washed with purified water. Afterward, the cuttings were inserted into a sterilized foam plate and immediately put into a hydroponic tank. One-half of the basal cut ends of the red- and green-stem cuttings were dipped in a 150 mg/L NAA solution, and the other half were dipped in pure water for 2 h. The concentration of NAA solution was determined according to agriculture practice. Any remaining residue was rinsed with pure water, then the cuttings were cultured in basic nutrient solution (1/10-strength Hoagland nutrient solution). Cuttings were grown in a greenhouse with 14/10 h (light/dark) and 60% relative humidity at 30/22 °C (day/night). In terms of the hydroponic system used, ventilation was performed by means of air pumps, and the water was changed once a week.

At 0, 1, 8, 15, 22, 29, 36, and 43 days, 162 stem cuttings (each replicate contained 54 samples) of each treatment were used to investigate the callus induction rate, rooting rate and death rate of the cuttings. Meanwhile, another batch of stem cuttings were collected on days 1, 8 and 15. Their basal parts (approximately 1.0 cm of the root zone) of the stem cuttings were harvested, flash frozen in liquid nitrogen and stored at − 80 °C for RNA-seq analysis and endogenous hormone content determination. All specimens above were morphologically identified by associate professor Jinbo Liang from Tea Research Institute of Enshi Academy of Agricultural Sciences,Enshi,China. The voucher specimens were deposited in the herbarium of Tea Research Institute of Enshi Academy of Agricultural Sciences,Enshi,China (Supplementary Table S5). Our field study and Experimental research complied with local legislation, national and international guidelines.

RNA-seq analysis and hormone content determination were repeated for three biological replicates. The detail RNA-seq sample information is listed in Table 1. To simplify the presentation, samples A, E, I represent red-stem cuttings (1, 8 and 15 days) under control. Samples B, F, J represent green-stem cuttings (1, 8 and 15 days) under control. Samples C, R, K represent red-stem cuttings (1, 8 and 15 days) under NAA treatment. Samples D, H, L represent green-stem cuttings (1, 8 and 15 days) under NAA treatment.

### Determination of endogenous hormone contents

High-performance liquid chromatography (HPLC)-mass spectrometry (MS)/MS analysis was performed to determine the contents of five endogenous hormones (IAA, ABA, GA1, GA3 and TZR) [54, 55]. The endogenous hormones were extracted by isopropanol/water/hydrochloric acid extraction. In brief, approximately 1.0 g of fresh plant samples was ground in liquid nitrogen until a powder was formed. Afterward, 10 mL of isopropanol/hydrochloric acid buffer was added to the powder, after which the mixture was incubated at 4 °C for 30 min under oscillation. After adding 20 mL of dichloromethane, the sample was further incubated at 4 °C for 30 min under oscillation. The mixture was then centrifuged at 13,000 r/min at 4 °C for 5 min, and the organic phase in the lower layer was collected. Nitrogen was used to air-dry the organic phase in the dark. Subsequently, the sediment was suspended in 400 μL of methanol (0.1% formic acid). After filtration through a 0.22 μm filter membrane, the hormones in the solution were measured via HPLC–MS/MS. The endogenous hormone contents were determined by an Agilent 1290 HPLC system coupled with a reversed-phase column (Poroshell 120 SB-C18, 2.1 × 150, 2.7 μm) maintained at 30 °C. Distilled water consisting of 0.1% formic acid was used as mobile phase A, and methanol consisting of 0.1% formic acid was used as mobile phase B. After separating the sample material by HPLC, an AB Qtrap6500 mass spectrometer with electrospray ionization (ESI) as the ion source and multi-channel detection (MRM) mode was used. After the parent ion is specifically selected by the mass analyzer, the parent ion is dissociated by collision induction in the collision reaction chamber so that this ion is cleaved to generate daughter ion. For most hormones, we detect two or three daughter ions and select the one with the highest response value as the quantitative ion (Supplementary Table S6).

### RNA extraction, library preparation and RNA-seq

Total RNA was extracted using a plant RNA extraction kit (Bioteke, China). RNA concentration and integrity were assessed using a Qubit 2.0 Fluorometer (Life Technologies, CA, USA) and an Agilent 2100 Bioanalyzer (Agilent Technologies, CA, USA). Each sample (3 µg) was used for sequencing library preparation using a NEBNext® Ultra™ RNA Library Prep Kit (NEB, USA) following the manufacturer’s instructions. The library quality was subsequently assessed via an Agilent 2100 Bioanalyzer system. An Illumina NovaSeq 6000 platform was used for subsequent sequencing.

### Data filtering and transcriptome analysis

After removing the adaptor sequences, ambiguous reads and low-quality reads, the remaining high-quality clean reads were used for the subsequent analysis. The clean reads were aligned to the tea plant genome (http://tpia.teaplant.org/) [56] using HISAT v2.0.5 software [57]. StringTie v1.3.3b software (http://ccb.jhu.edu/software/stringtie) was used to predict the new genes [58]. ClusterProfile software was used for GO (Gene Ontology) functional analysis, and the KEGG (Kyoto Encyclopedia of Genes and Genomes) (https://www.kegg.jp/kegg/kegg1.html) database was utilized for the pathway enrichment analysis. OmicShare tools (http://www.omicshare.com/tools) were used to graphically display the results of the GO and KEGG functional enrichment analyses, with adjusted *p* values < 0.05 constituting the threshold. WGCNA was performed to construct gene co-expression networks [59]. The networks were subsequently established and visualized with Cytoscape 3.5.1 [60].

### Identification of DEGs

Fragments per kilobase of transcript per million fragments (FPKM) values were used to normalize gene expression. DESeq2 R software (1.16.1) was used to identify differentially expressed genes (DEGs, NAA vs. CK) according to the following threshold criteria: |log2(fold change)|> 1, *p*-adjusted < 0.05 [61]. Correlations of biological repeats were evaluated using Pearson’s correlation coefficient [62].

### Quantitative real-time PCR verification analysis

To validate the accuracy of the RNA-seq results, 8 DEGs were chosen for validation using reverse transcription quantitative real-time PCR (RT-qPCR). One microgram of total RNA was used to synthesize first-strand cDNA using a PrimeScript™ RT Reagent Kit together with gDNA Eraser according to the manufacturer’s instructions (TaKaRa, Dalian, China). RT-qPCR was performed in a 20 μL reaction mixture consisting of 10 µL of SYBR Premix Ex, 2 µL of diluted template cDNA, 0.4 µL of each primer, and 7.2 μL of ddH_2_O. RT-qPCR was conducted on a 7500 Real-Time PCR System (Applied Biosystems, Waltham, MA, USA) with the following thermocycling programme: denaturation at 95 °C for 30 s, followed by 40 cycles of 95 °C for 10 s, 58 °C for 15 s and 72 °C for 12 s. All the primers used in this assay are listed in Table 3, and the tea plant *GAPDH* gene was used as a reference [63]. Each reaction was performed in three biological repeats, and the relative gene expression levels were calculated using the 2^−∆∆CT^ method [64].

**Table 3:** Sequences of primers used for RT-qPCR verification

Gene name	Description	Primer sequence(5’-3’)
TEA008965	Aquaporin protein AQU21	F: CTTCCTCGGTGGCAACATAA
		R: ATCAACTGCCGTGGCGTAG
TEA004281	Beta-glucosidase 12	F: TGGGATACCTTCACGCATAGA
		R: CTTGTTCACTCCACCACTTAGCT
novel.4690	Cellulose synthase-like protein H1	F: GAGGTTGGCTGGATGTATGGA
		R: GAAATGCAGGTGGGTTAGGTG
TEA007001	Cytochrome P450	F: ATTGGAGGCAAATGAGAAAGG
		R: GCAGATTGTGGTGCTTGTGAG
TEA018671	U-box domain-containing protein 19	F: GGGGCTTCTGAGCAGTCTAATT
		R: GGTCGATACGGTCACAGGGT
novel.10242	Aldose 1-epimerase	F: ACCCGTAGAAACCCATTCACTC
		R: CTCTTCAGCTCATAAATCCCAACA
TEA017805	Sulfate transporter 3.3	F: GATTTCCTCGTGTTGTTATGTGC
		R: TCTGGCAGCCTCTTTGTATTGT
TEA014068	Zinc finger CCCH domain-containing protein 49	F: GCTATGGAGGGTTCTTGTGCT
		R: GAGAAGCCACCATTTCGTTGA
*GAPDH*	Glyceraldehyde-3-phosphate dehydrogenase	F: TTGGCATCGTTGAGGGTCT
		R: CAGTGGGAACACGGAAAGC

## Supplementary Information

**Additional file 1: Figure S1.** Effects of NAA treatment on endogenous hormone changes in red- and green-stem cuttings of tea plant. **a** GA1 contents. **b** GA3 contents. R-CK, control group of red-stem cuttings; R-NAA, NAA treatment group of red-stem cuttings; G-CK, control group of green-stem cuttings; G-NAA, NAA treatment group of green-stem cuttings.

**Additional file 2: Figure S2.** Correlations of gene expression levels between measured samples.

**Additional file 3: Figure S3.** GO enrichment analysis of DEGs from red (**a**)- and green (**b**)-stem cutting groups.

**Additional file 4: Figure S4.** KEGG enrichment analysis of DEGs from red (**a**)- and green (**b**)-stem cutting groups.

**Additional file 5: Table S1.** KEGG enrichment analysis of DEGs in both the red- and green-stem cutting groups.

**Additional file 6: Table S2.** KEGG enrichment analysis of DEGs in the red-stem cutting groups.

**Additional file 7: Table S3.** KEGG enrichment analysis of DEGs in the green-stem cutting groups.

**Additional file 8: Table S4.** Hormone-related DEGs in both the red- and green-stem cutting groups.

**Additional file 9: Table S5.** Locality and voucher information for the tea cutting samples.

**Additional file 10: Table S6.** Selected reaction monitoring conditions for protonated or deprotonated plant hormones ([M+H]^+^or[M-H]^-^)

## Data Availability

The sequence data have been submitted to the NCBI Sequence Read Archive (http://trace.ncbi.nlm.nih.gov/Traces/sra/) under BioProject accession number PRJNA690632.

## Abbreviations

ABA: Abscisic acid
AR: Adventitious root
ARF: Auxin response factor
BR: Brassinosteroid
DEG: Differentially expressed gene
ET: Ethylene
FPKM: Fragments per kilobase of transcript per million fragments
GA: Gibberellin
GO: Gene Ontology
HPLC: High-performance liquid chromatography
IAA: Indole-3-acetic acid
IBA: Indole-3-butyric acid
KEGG: Kyoto Encyclopedia of Genes and Genomes
NAA: Naphthaleneacetic acid
SAUR: SMALL AUXIN UP RNA
WGCNA: Weighted gene co-expression network analysis
