# Supplementary figures and images for: Integrated transcriptome and hormonal analysis of naphthalene acetic acid-induced adventitious root formation of tea cuttings (Camellia sinensis)
Source: BMC Plant Biol. 2022 Jul 4;22:319. doi: 10.1186/s12870-022-03701-x (PMC9251942; doi:10.1186/s12870-022-03701-x)

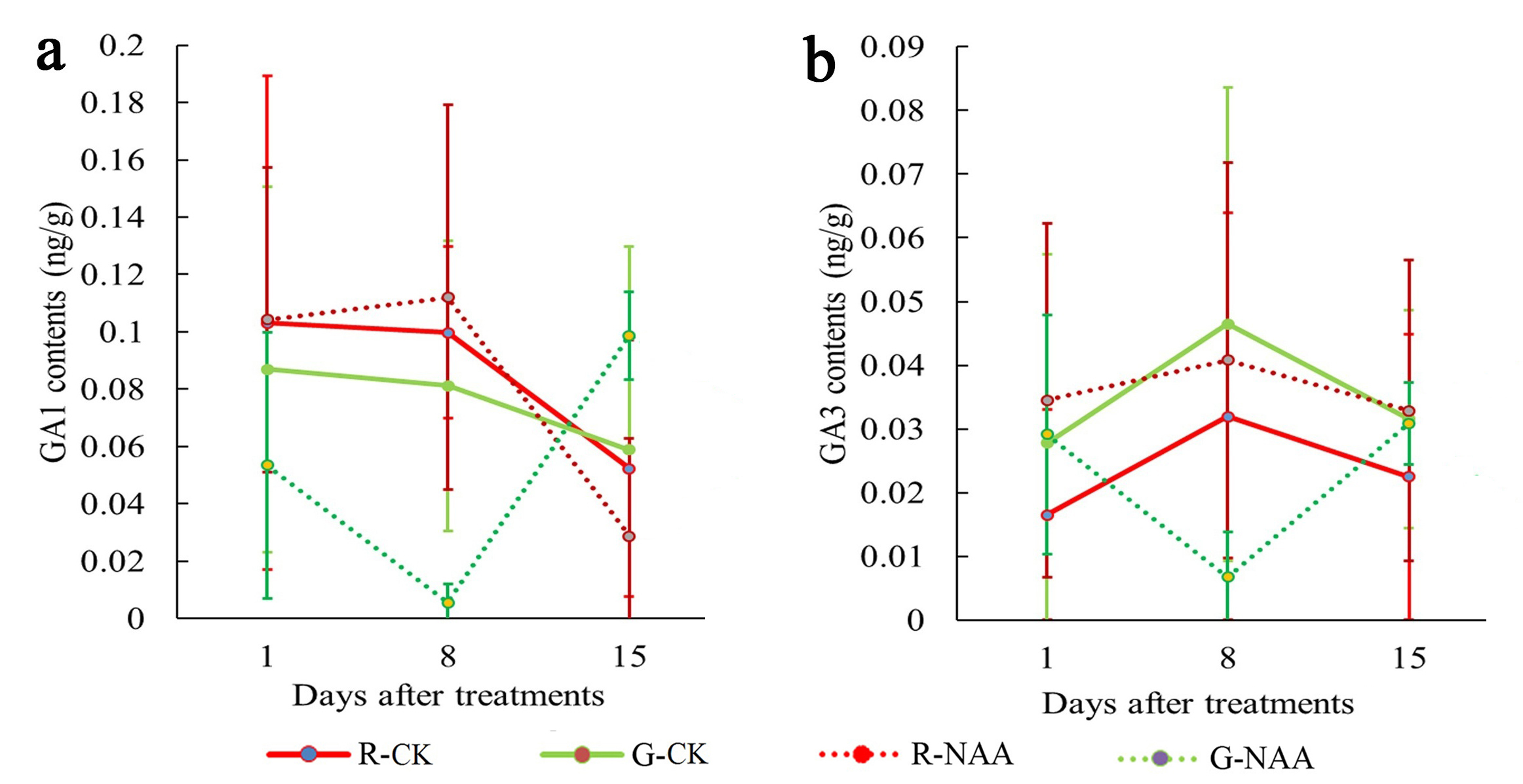

Supplement: Supplementary file 1 — Additional file 1: Figure S1. Effects of NAA treatment on endogenous hormone changes in red- and green-stem cuttings of tea plant. a GA1 contents. b GA3 contents. R-CK, control group of red-stem cuttings; R-NAA, NAA treatment group of red-stem cuttings; G-CK, control group of green-stem cuttings; G-NAA, NAA treatment group of green-stem cuttings. [file 12870_2022_3701_MOESM1_ESM.jpg]

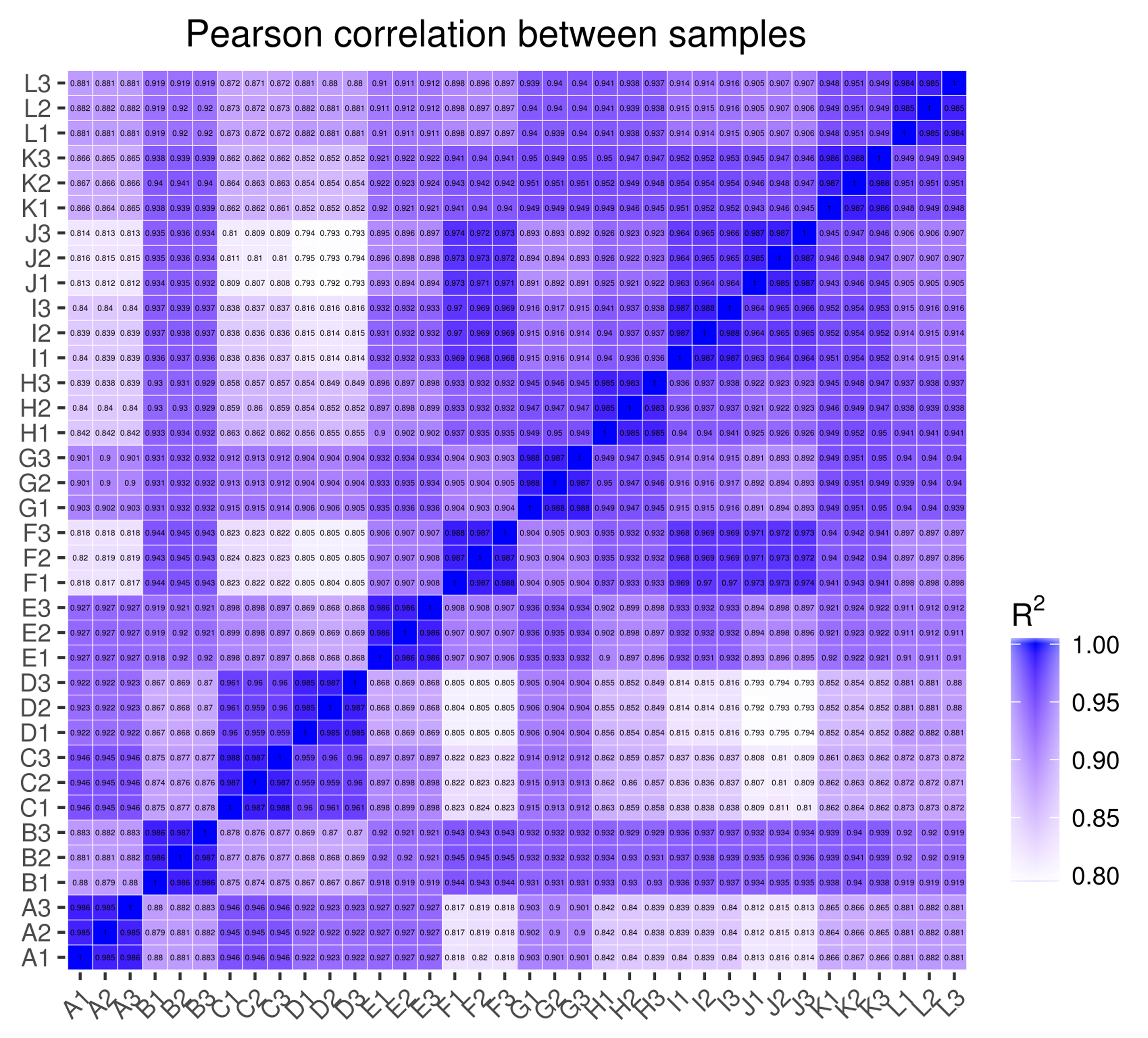

Supplement: Supplementary file 2 — Additional file 2: Figure S2. Correlations of gene expression levels between measured samples. [file 12870_2022_3701_MOESM2_ESM.jpg]

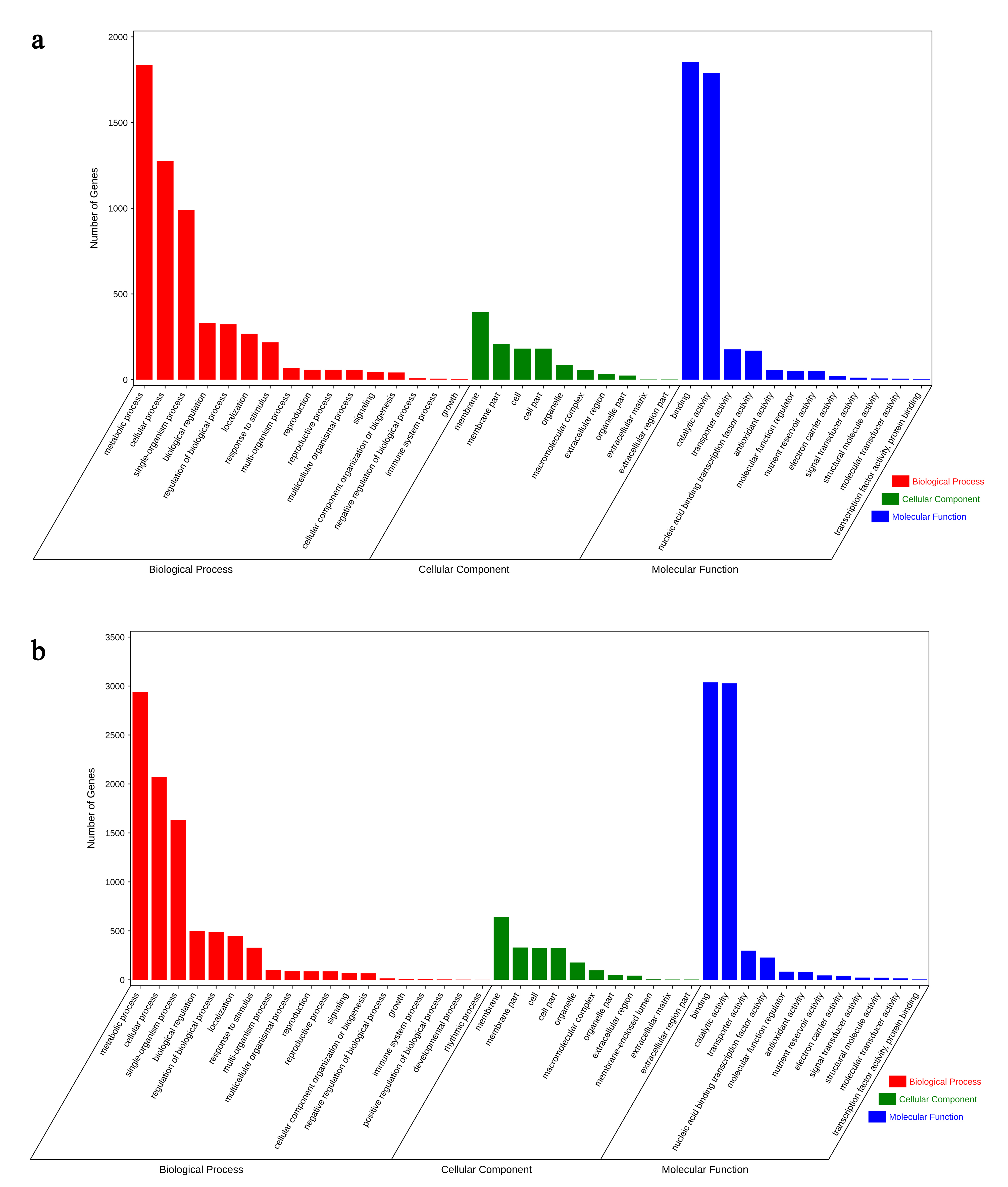

Supplement: Supplementary file 3 — Additional file 3: Figure S3. GO enrichment analysis of DEGs from red (a)- and green (b)-stem cutting groups. [file 12870_2022_3701_MOESM3_ESM.jpg]

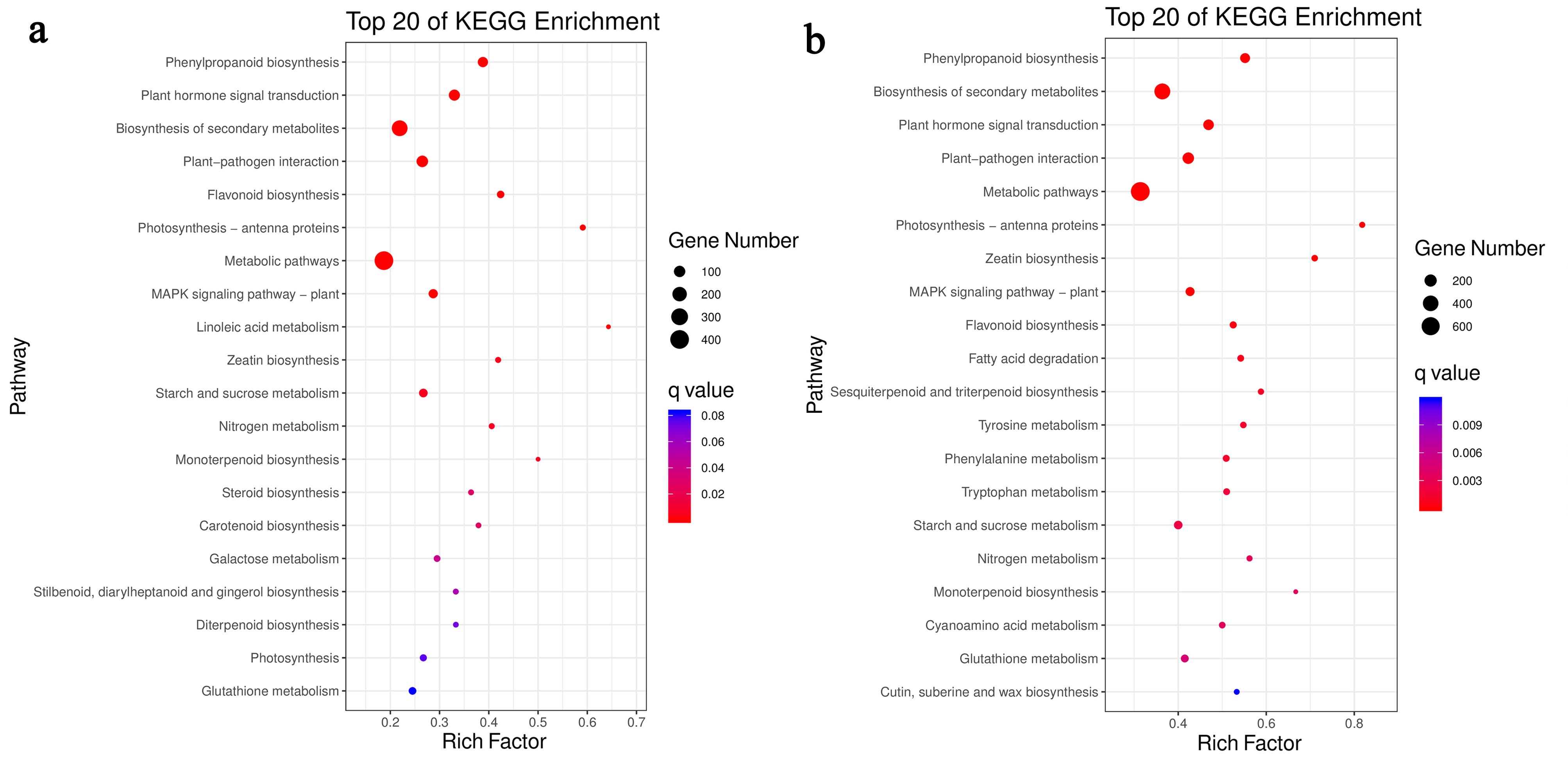

Supplement: Supplementary file 4 — Additional file 4: Figure S4. KEGG enrichment analysis of DEGs from red (a)- and green (b)-stem cutting groups. [file 12870_2022_3701_MOESM4_ESM.jpg]
