# Supplementary material for: Integrated transcriptome and hormonal analysis of naphthalene acetic acid-induced adventitious root formation of tea cuttings (Camellia sinensis)
Source: BMC Plant Biol. 2022 Jul 4;22:319. doi: 10.1186/s12870-022-03701-x (PMC9251942; doi:10.1186/s12870-022-03701-x)
Supplement: Supplementary file 9 — Additional file 9: Table S5. Locality and voucher information for the tea cutting samples. [file 12870_2022_3701_MOESM9_ESM.docx]

**Table S5. Locality and voucher information for the tea cutting samples**

| **Species** | **Voucher no.** | **Collection locality** | **Geographic coordinates** | **Sample sizes** |
| --- | --- | --- | --- | --- |
| *C. sinensis* (L.) O. Kuntze | 2,018,715-0D(1 ~ 12) | Enshi | 30°11′ 47″ N and 109° 23′ 36″ E | 12 |
|  | 2,018,716-1D(13 ~ 24) | Enshi | 30°11′ 47″ N and 109° 23′ 36″ E | 12 |
|  | 2,018,723-8D(25 ~ 36) | Enshi | 30°11′ 47″ N and 109° 23′ 36″ E | 12 |
|  | 2,018,730-15D(37 ~ 48) | Enshi | 30°11′ 47″ N and 109° 23′ 36″ E | 12 |
|  | 2,018,806-22D(49 ~ 60) | Enshi | 30°11′ 47″ N and 109° 23′ 36″ E | 12 |
|  | 2,018,813-29D(61 ~ 72) | Enshi | 30°11′ 47″ N and 109° 23′ 36″ E | 12 |
|  | 2,018,820-36D(73 ~ 84) | Enshi | 30°11′ 47″ N and 109° 23′ 36″ E | 12 |
|  | 2,018,827-43D(85 ~ 96) | Enshi | 30°11′ 47″ N and 109° 23′ 36″ E | 12 |
